# A Novel Unified Framework for Energy-Based Spectrum Sensing Analysis in the Presence of Fading

**DOI:** 10.3390/s22051742

**Published:** 2022-02-23

**Authors:** Aleksey S. Gvozdarev

**Affiliations:** Intelligent Information Radiophysics Systems Department, P. G. Demidov Yaroslavl State University, 150003 Yaroslavl, Russia; asg.rus@gmail.com

**Keywords:** fading channel, Beaulieu-Xie shadowed, Fluctuating Beckmann, area under the curve, probability of detection

## Abstract

This paper studies the performance of the energy-based sensing procedure in the presence of multipath fading and shadowing effects in terms of its average probability of detection (APD), average receiver operating characteristic (AROC) and the area under the AROC curve (AUC). A new generalization for the class of the fading channel moment generating functions (MGFs) (i.e., factorized power type (FPT) MGF) was proposed and applied for the construction of the unified framework for the analytical treatment of the formulated problem. The contiguity of the proposed model with the existing classical ones (Rayleigh, Nakagami-m, Hoyt, η−μ, κ−μ shadowed and Mixture-Gamma) was demonstrated. Within the assumed MGF representation, the novel closed-form solutions and computationally efficient approximation for APD and AUC are derived. The obtained general expressions were then applied for derivation of the new results for the recent generalized fading channel models: Fluctuating Beckmann and Beaulieu-Xie shadowed. For each of the models, high-SNR asymptotic expressions were obtained. Lastly, numeric simulation was performed to verify the correctness of the derived results, to establish the dependencies of the sensing performance quality from the channel parameters and to identify the specific ranges of their asymptotic behavior.

## 1. Introduction

Currently, the implementation of cognitive principles in internet of things (IoT) applications and various ad hoc communication systems leads to the simplification of involved mobile devices (M2M, D2D communications, etc.), and thus to the requirement of computational load reduction. It leads to the fact that in most cases energy-based detection (ED) [1], as one of the simplest and most straightforward methods for the practical implementation of the white-space detection strategies [2], is favored. Although the most general ED quality description is well known since the pioneering work of [3] and is given in terms of the average probability of detection (APD), average receiver operating characteristic (AROC) and the area under the AROC curve (AUC) (see [1]), the exact expressions for a specific communication system fully depend on the assumed microwave wireless propagation channel model, which incorporates such effects as multipath fading and shadowing. For modern communication systems, the increased amount of communicating mobile machines and devices leads to the contraction of the coverage area (for instance, as in 5G) and the impairment of the signal-to-noise/interference (SNR/SINR) environments. Thus, the existing classical wireless channel fading models (i.e., Rayleigh, Rician, Nakagami-m, Hoyt, etc.) do not fully comply with real-life measurements.

The solution of the problem is usually sought involving the so-called generalized channel models [4,5,6] (such as η−μ, κ−μ, Generalized Gamma, Fading Beckmann, etc.), that inherently include as specific limiting cases the simplified classical models (Rayleigh, Rician, Nakagami-m, Hoyt, etc.). In most cases, their flexibility is obtained at the expense of the higher analytic and computational complexity [7]. However, from a practical perspective, it is highly desired for scientific researchers and engineers to have at hand closed-form expressions for ROC/AUC that can be used for the design of efficient optimization strategies in terms of link quality and reliability.

Numerous studies have proposed a variety of forms of representation for the ROC, AROC and AUC for several generalized channel models (as well as for the majority of simplified models) [1,8,9,10,11,12,13,14,15,16,17], but in many cases, they do not yield closed-form expressions and are presented in terms of infinite series of hypergeometric functions of multiple variables (such as H-Fox function) (see, for instance, [14,18]), multidimensional nested infinite series (see, for instance, [19]) or parametric integrals, which are of limited usefulness for further generalization and practical implementation, or assume only integer-valued fading parameters [20], restricting their applicability. Moreover, the approaches that were used to derive those solutions were generally quite isolated and the obtained representations had dissimilar mathematical structures and notations. Over the years, several attempts have been made for the unification of the analytical solution of the problem. For instance, ref. [21,22] proposed a solution by exploiting the exponential-type integral for the Marcum Q-function and demonstrated numerical results for η−μ, κ−μ and Nakagami-m fading channels. Although the derived result is claimed to be uniformly applicable (if the MGF expression is at hand) no closed-form solutions were presented for the specific models. Moreover, the existing representation was valid for the integer sensing base (which is a common limitation for most of the solutions) and had to be performed numerically. Almost in parallel, ref. [23] proposed a similar solution with exactly the same drawbacks and limitations. The more sophisticated and promising approach was proposed in an excellent paper [24], where the average probability of a false alarm was derived in terms of the Mellin transform of the instantaneous SNR probability density function and exponential-type integral representation of the Marcum Q-function, which eventually led to the solutions (obtained in terms of multivariate Fox H-functions) for Rayleigh, Maxwell, Nakagami-m, Weibull, Generalized Gamma and EKG fading channels. Although a substantial step towards the unification of the derived closed-form results was achieved, it possesses several drawbacks, including analytical, as even though some results were derived it was still hard to predict the solutions for the similar types of fading distributions, and numerical, as, for high APDs, the solution was subjected to numerical underflow and thus only asymptotic expressions were used in those cases.

Motivated by the above works, the present research studies the possible construction of the unified framework for the analytical treatment of energy-based sensing in the presence of multipath fading. The major contributions of this work can be summarized as follows:

- For the unification of the wide range of existing channel models, a new moment-generating function model (i.e., the factorized power-type (FPT) representation) was introduced. It is demonstrated that such a generalization can easily handle non-line-of-sight and shadowed line-of-sight models widely applied in communication theory.
- Under this assumption, applying the contour-integral transformation technique, closed-form analytic expressions for the average probability of detection and area under the receiver operating characteristic curve are derived, and their simple interconnections are established.
- Based on the high signal-to-noise ratio assumption, asymptotic expressions for the FTP models’ APD and AUC, useful for numeric computation, are derived.
- Capitalizing on the obtained results, the novel closed-form representations of the aforementioned detection quality metrics and their asymptotic versions for the Fluctuating Beckmann and the Beaulieu-Xie shadowed models were evaluated. Lastly, the validating numeric simulation was executed to establish the dependencies of the sensing performance from the channel parameters and identify the ranges of their asymptotic behavior.

The remainder of the paper is organized as follows: Section 2 provides some preliminaries that include the formal definition of the energy-based detection procedure and its quality metrics; Section 3 introduces the model of the FPT MGF, demonstrates how it degenerates into most widely used simplified cases (Rayleigh, Nakagami-m, Hoyt, η−μ, κ−μ shadowed, Mixture-Gamma), derives the closed-form and asymptotic expressions of the APD and AUC for the FTP model and applies them to obtain novel results for the Fluctuating Beckmann and Beaulieu-Xie shadowed channel models; Section 4 presents some numerical results that demonstrate the correctness of the proposed solutions and analyzes the performance of energy-based detection quality for the Fluctuating Beckmann and Beaulieu-Xie shadowed channel models depending on the parameter values; Section 5 discusses possible generalizations and extensions of the proposed results and computational aspects; and conclusions are drawn in Section 6.

## 2. Preliminaries

The classical energy-based detection of an unknown deterministic signal in the presence of additive white Gaussian noise is usually regarded as a hypothesis testing problem, quantitatively described [1] by the probability of detection $P_{D}$ and false alarm $P_{F}$ and expressed in terms of the generalized Marcum-Q function $Q_{u}\left(a,b\right)$ and the regularized upper incomplete Gamma function $\tilde{\Gamma }\left(a,b\right)$ [25]:

(1) $$\left\{\begin{array}{c} P_{D}=Q_{u}\left(\sqrt{2\gamma },\sqrt{\lambda }\right),\\ P_{F}=\tilde{\Gamma }\left(u,\frac{\lambda }{2}\right), \end{array}\right.$$

where λ is the decision threshold.

For a wireless multipath fading channel with the probability density function of the instantaneous SNR $w_{\gamma }\left(\gamma \right)$, the averaged probability of detection is given by

(2) $$\begin{array}{c} {\bar{P}}_{D}=\int_{0}^{\infty }P_{D}\left(\gamma \right)w_{\gamma }\left(\gamma \right)d\gamma . \end{array}$$

Evaluating λ for a fixed level of $P_{F}$ as $\lambda =2{\tilde{\Gamma }}^{-1}\left(u,P_{F}\right)$ (where ${\tilde{\Gamma }}^{-1}\left(\cdot ,\cdot \right)$ is the inverse regularized upper incomplete Gamma function [25]), substituting into (2) and integrating over the whole range yields the expression for the AUC:

(3) $$\begin{array}{c} \;\mathrm{AUC}\;=\;\int_{0}^{1}\;{\bar{P}}_{D}\;\left(P_{F}\right){dP}_{F}. \end{array}$$

The main problem is that (2) and (3) have closed-form solutions only for a limited number of channel models. Moreover, the known results are too diversiform in notation and could not be straightforwardly unified and generalized.

## 3. Derived Results

### 3.1. Channel Model with FPT MGF

To solve the aforementioned problem, let us define a generalized fading channel model with the MGF (i.e., $M_{\gamma }\left(s\right)=E\left\{e^{\gamma s}\right\}$) that has a factorized power-type representation as follows:

(4) $$\begin{array}{c} M_{\gamma }^{\mathrm{FPT}}\left(s\right)≜A_{p}\prod_{j=1}^{N}\delta_{j}{\left(\alpha_{j}-s\right)}^{\beta_{j}}, \end{array}$$

with a set of coefficients $A_{p},\delta_{j},\alpha_{j},\beta_{j},N$ (it should be emphasized that this definition is close to the monomial/posynomial MGF defined in [26] (see Section 5 for discussion)). It can be seen that defining those coefficients in a specific way reduces (4) to various simplified models, including Rayleigh, Nakagami-m, Hoyt, η−μ, κ−μ shadowed, Gamma, Mixture-Gamma, etc. Moreover, one can notice that those models encompass non-line-of-sight (NLoS) and shadowed line-of-sight models, hence covering a wide range of possible applications. It can be observed that $A_{p}\prod_{j=1}^{N}\delta_{j}\alpha_{j}^{\beta_{j}}=M_{\gamma }\left(0\right)=1$, hence, for most of the further results, such a multiplier is omitted.

### 3.2. Special Simplified Cases of the FPT MGF Model. Models’ Connections

In general, as was stated, (4) encompasses a wide variety of the existing fading channel models. Thus, *N* can be assumed as a parameter defining the order of the model, the normalizing coefficient $A_{p}$ and coefficients $\alpha_{j},\forall j=\bar{1,N}$ depend on the average signal-to-noise ratio $\bar{\gamma }$ (usually being inversely proportional to $\bar{\gamma }$, i.e., αj∼1γ¯) and channel-specific parameters: the number of multipath clusters, number of line-of-sight (LoS) components, shadowing intensity, amount of energy within the dominant components, relative to the multipath waves, total energy in dominant components etc. Coefficients $\beta_{j},\forall j=\bar{1,N}$ are described in terms of channel parameters only. The coefficients $\delta_{j}$ are specific numerical multiplicative coefficients (in most cases equal to unity). Let us define the interrelations between the channel physical parameters and coefficients in (4) for the specific channel models.

- Rayleigh. The Rayleigh fading channel model is the classical one and is among the most frequently used in cases of NLoS situations. In can be seen that to be in full compliance with the Rayleigh MGF, defined, for instance, as in [4], one has to perform the following set of substitutions in (4): N=1, Ap=1γ¯, $\delta_{1}=1$, α1=1γ¯, $\beta_{1}=-1$.
- Nakagami-m. The Nakagami-m fading channel is usually assumed to be more versatile than the Rayleigh model, including scenarios with fading that is heavier and lighter than Rayleigh. Following the same procedure as before and matching the definition of the Nakagami-m MGF (given, for example, in [4]) with (4), the substitutions will look like: N=1, Ap=mγ¯m, $\delta_{1}=1$, α1=mγ¯, $\beta_{1}=-m$.
- Hoyt. The Hoyt fading channel model distribution is typically employed to model the enriched multipath fading [17] (for instance, in cases of strong ionospheric scintillation in satellite links or mobile satellite channels being simulated in the form of a two-state process). Contrary to Rayleigh and Nakagami-m, the application of the Hoyt MGF definition given in [4] leads to N=2, δ1,2=(−1)12, and other parameters will be Ap=−(1+q2)2γ¯q, α1=(1+q2)2γ¯, α2=(1+q2)2γ¯q2, β1,2=−12.
- η−μ. The η−μ is usually the extension of the abovementioned models, thus matching the definition, given, for example, in [5], with (4) leading to the following substitutions: N=2, $A_{p}={\left(\frac{{\bar{\gamma }}^{2}}{4\mu^{2}\left(h^{2}-H^{2}\right)}\right)}^{-\mu }$, $\delta_{1,2}=1$, $\alpha_{1}=\frac{2\mu (h-H)}{\bar{\gamma }}$, $\alpha_{2}=\frac{2\mu (h+H)}{\bar{\gamma }}$, $\beta_{1,2}=-\mu$.
- κ−μ shadowed. The κ−μ shadowed fading channel model, that has recently drawn much attention [27], was first presented in [28], and defines a generalized model accounting for the most of the abovementioned cases (with the exception of the Hoyt model, see [6]) combined with the shadowed LoS situation. The parameters $\bar{\gamma },m,\kappa ,\mu$, defined as in [28] and connected with (4), are as follows: N=2, $A_{p}=\frac{{\left(-\mu \right)}^{\mu }m^{m}{\left(1+\kappa \right)}^{\mu }}{{\bar{\gamma }}^{\mu }{\left(\mu \kappa +m\right)}^{m}}$, $\delta_{1,2}=1$, $\alpha_{1}=\frac{\mu (1+\kappa )}{\bar{\gamma }}$, $\alpha_{2}=\frac{\mu (1+\kappa )}{\bar{\gamma }}\frac{m}{\left(\mu \kappa +m\right)}$, $\beta_{1}=m-\mu$, $\beta_{2}=-m$.
- Mixture-Gamma. Amidst the existing channel models, Mixture-Gamma stands out and is regarded as having paramount importance, since it can successively approximate a wide a range of the existing models, including the aforementioned ones and their generalizations (see [11]). The principal difference between (4) and the Mixture-Gamma MGF (defined in [11] in terms of parameters ${\tilde{\alpha }}_{n},{\tilde{\beta }}_{n},\zeta_{n}$) is that the latter can be viewed as a linear combination of $N_{p}$ versions of (4) with a specific treatment of normalization constants (i.e., $\sum_{n=1}^{N_{p}}\frac{{\tilde{\alpha }}_{n}\Gamma \left({\tilde{\beta }}_{n}\right)}{{\left(\zeta_{n}\right)}^{{\tilde{\beta }}_{n}}}=1$). For each of the $N_{p}$ summands, the substitutions are as follows: N=1, $A_{p}={\tilde{\alpha }}_{n}\Gamma \left({\tilde{\beta }}_{n}\right)$, $\delta_{1}=1$, $\alpha_{1}=\zeta_{n}$, $\beta_{1}=-{\tilde{\beta }}_{n}$. Moreover, ref. [29] states Mixture-Gamma as an approximation model for such composite fading channels as κ−μ/Gamma, η−μ/Gamma and α−μ/Gamma, thus expanding the applicability of the proposed FPT MGF model.

### 3.3. General Results

Capitalizing on the proposed FPT MGF model, it is possible to derive closed-form solutions for (2) and (3).

**Theorem** **1.** *For energy-based detection in the presence of a multipath fading channel with the factorized power-type moment generating function, the average probability of detection and area under the receiver operating characteristic curve are given by*

(5) P¯DFPT=Ap∏j=1Nδjαjβj1−λλ22ue−λ/2Γ(u+1)∏j=1N1+1αjβjΦ2(N+1)1,−β1,…,−βN;u+1;λ2,λ21+α1,…,λ21+αN

(6) AUCFPT=Ap∏j=1Nδjαjβj1−∏j=1N1+1αjβjΓu+122πΓu+1FD(N+1)2u;1,−β1,…,−βN;u+1;12,121+α1,…,121+αN

**Proof** **of** **Theorem** **1.** For proof, see Appendix A. □

As was noted above, from the connections of the FPT MGF model with classical ones, it can be easily verified that the parameters $\alpha_{j}$ are usually inversely proportional to the SNR (instantaneous or average), thus the case when $\alpha_{j}\to 0$ can be regarded as the high-SNR regime. In such a situation, some simplifications of the general solutions (5) and (6) can be derived.

**Theorem** **2.** *The asymptotic versions of the APD and AUC for the FPT MGF model (for the case of*$\alpha_{i}\to 0\;\;\forall i\in \left[1,N\right]$) *are given by*

(7) P¯DasFPT≃Ap∏j=1Nδjαjβj1−∏j=1N1+1αjβjλλ22ue−λ/2Γu+11F11−∑i=1Nβi;u+1;λ2+

(8) $$\begin{array}{c} {\mathrm{AUC}}_{\mathrm{as}}^{\mathrm{FPT}}≃\left(A_{p}\prod_{j=1}^{N}\delta_{j}\alpha_{j}^{\beta_{j}}\right){\left[1-\prod_{j=1}^{N}{\left(1+\frac{1}{\alpha_{j}}\right)}^{\beta_{j}}\frac{\Gamma \left(u+\frac{1}{2}\right)}{2\sqrt{\pi }\Gamma \left(u+1\right)}{}_{2}F_{1}\left(2u,1-\sum_{i=1}^{N}\beta_{i};u+1;\frac{1}{2}\right)\right]}_{+} \end{array}$$

*where*${}_{1}F_{1}\left(\cdot \right)$*is the confluent hypergeometric function,*${}_{2}F_{1}\left(\cdot \right)$*is the Gauss hypergeometric function and*${\left[a\right]}_{+}$*is the positive part of a, i.e.,*${\left[a\right]}_{+}=\max\left(0,a\right)$.

**Proof** **of** **Theorem** **2.** For proof, see Appendix B. □

It should be specifically noted that, contrary to several adopted approaches, the proposed one and hence the derived results are valid for arbitrary values of channel parameters and sensing base, which in most studies are assumed to be integer-valued.

As was stated, the model (4) generalizes a wide range of intensively used simplified channel models. Hence, Theorems 1 and 2 provide a solid ground for the unification of the derived quality metrics (APD and AUC) of those models. The APD and AUC expressions for the models included in Section 3.2, that can be derived directly from (5) and (6), with some exceptions, were generally reported in the literature, but in differing forms (see [8,16] for Rayleigh, [8] for Nakagami-m, [17] for Hoyt, [9,13] for η−μ, [14,30,31] for κ−μ shadowed) and hence are of limited interest, mainly for illustrative purposes and notation unification.

On the other hand, Theorems 1 and 2 can be applied to the recently proposed channel models for which the results are not present. Among them, one can focus upon the two novel generalized models: Fluctuating Beckmann (see [32,33,34,35,36]) and Beaulieu-Xie shadowed (see [37,38,39,40]).

### 3.4. Application of the Derived Results

#### 3.4.1. Exact and Asymptotic APD and AUC for the Fluctuating Beckmann Channel Model

For the Fluctuating Beckmann fading channel model, the MGF is given by

(9) $$\begin{array}{c} M_{\gamma }^{\mathrm{FB}}\left(s\right)=\frac{{\left(-1\right)}^{\mu }}{p^{\mu }}\frac{\alpha_{2}^{m-\frac{\mu }{2}}}{{\bar{\gamma }}^{\mu }\alpha_{1}^{m}}{\left(1-\frac{\mu (1+\eta )(1+\kappa )}{2\eta \bar{\gamma }p}\right)}^{m-\frac{\mu }{2}}\times \\ \times {\left(1-\frac{\mu (1+\eta )(1+\kappa )}{2\bar{\gamma }p}\right)}^{m-\frac{\mu }{2}}\;{\left(1-\frac{c_{1}}{\bar{\gamma }p}\right)}^{-m}\;{\left(1-\frac{c_{2}}{\bar{\gamma }p}\right)}^{-m}\;, \end{array}$$

with the parameters $\bar{\gamma },\eta ,m,\kappa ,\mu ,c_{1},c_{2}$ defined as in [32] and connected with (4) as follows: N=4, $A_{p}=\frac{\alpha_{2}^{m-\mu }}{{\bar{\gamma }}^{\mu }\alpha_{2}^{m}}$, $\delta_{1-4}=1$, $\alpha_{1}=\frac{\mu (1+\kappa )(1+\eta )}{2\eta \bar{\gamma }}$, $\alpha_{2}=\alpha_{1}\eta$, $\alpha_{3}=c_{1}$, $\alpha_{4}=c_{2}$, $\beta_{1,2}=m-\frac{\mu }{2}$, $\beta_{3,4}=-m$.

**Corollary** **1.** *The APD and AUC for this model in the most general form are given by*

(10) $$\begin{array}{c} {\bar{P}}_{D}^{\mathrm{FB}}=1-\frac{{\left(\frac{\lambda }{2}\right)}^{u}e^{-\frac{\lambda }{2}}}{\Gamma \left(u+1\right)}\frac{{\left(1+\frac{2\eta \bar{\gamma }}{\mu \left(1+\eta \right)\left(1+\kappa \right)}\right)}^{m-\frac{\mu }{2}}{\left(1+\frac{2\bar{\gamma }}{\mu \left(1+\eta \right)\left(1+\kappa \right)}\right)}^{m-\frac{\mu }{2}}}{{\left(1+\frac{\bar{\gamma }}{c_{1}}\right)}^{m}{\left(1+\frac{\bar{\gamma }}{c_{2}}\right)}^{m}}\times \\ \times \Phi_{2}^{\left(5\right)}\left(1,\frac{\mu }{2}-m,\frac{\mu }{2}-m,m,m;u+1;\frac{\lambda }{2},\frac{\lambda }{2}\left(\frac{2\eta \bar{\gamma }}{2\eta \bar{\gamma }+\mu \left(1+\eta \right)\left(1+\kappa \right)}\right),\frac{\lambda }{2}\left(\frac{2\bar{\gamma }}{2\bar{\gamma }+\mu \left(1+\eta \right)\left(1+\kappa \right)}\right),\frac{\lambda }{2}\left(\frac{\bar{\gamma }}{\bar{\gamma }+c_{1}}\right),\frac{\lambda }{2}\left(\frac{\bar{\gamma }}{\bar{\gamma }+c_{2}}\right)\right), \end{array}$$

(11) AUCFB=1−Γu+122πΓu+11+2ηγ¯μ1+η1+κm−μ21+2γ¯μ1+η1+κm−μ21+γ¯c1m1+γ¯c2m××FD(5)2u,1,μ2−m,μ2−m,m,m;u+1;12,122ηγ¯2ηγ¯+μ1+η1+κ,122γ¯2γ¯+μ1+η1+κ,12γ¯γ¯+c1,12γ¯γ¯+c2.

*and their asymptotic versions can be represented as*

(12) $$\begin{array}{c} {\bar{P}}_{D\mathrm{as}}^{\mathrm{FB}}≃{\left[1-\frac{{\left(1+\frac{2\eta \bar{\gamma }}{\mu \left(1+\eta \right)\left(1+\kappa \right)}\right)}^{m-\frac{\mu }{2}}{\left(1+\frac{2\bar{\gamma }}{\mu \left(1+\eta \right)\left(1+\kappa \right)}\right)}^{m-\frac{\mu }{2}}}{{\left(1+\frac{\bar{\gamma }}{c_{1}}\right)}^{m}{\left(1+\frac{\bar{\gamma }}{c_{2}}\right)}^{m}}\frac{{\left(\frac{\lambda }{2}\right)}^{u}e^{-\frac{\lambda }{2}}}{\Gamma \left(u+1\right)}{}_{1}F_{1}\left(\mu +1;u+1;\frac{\lambda }{2}\right)\right]}_{+} \end{array}$$

(13) $$\begin{array}{c} {\mathrm{AUC}}_{\mathrm{as}}^{\mathrm{FB}}≃{\left[1-\frac{{\left(1+\frac{2\eta \bar{\gamma }}{\mu \left(1+\eta \right)\left(1+\kappa \right)}\right)}^{m-\frac{\mu }{2}}{\left(1+\frac{2\bar{\gamma }}{\mu \left(1+\eta \right)\left(1+\kappa \right)}\right)}^{m-\frac{\mu }{2}}}{{\left(1+\frac{\bar{\gamma }}{c_{1}}\right)}^{m}{\left(1+\frac{\bar{\gamma }}{c_{2}}\right)}^{m}}\frac{\Gamma \left(u+\frac{1}{2}\right)}{2\sqrt{\pi }\Gamma \left(u+1\right)}{}_{2}F_{1}\left(2u,\mu +1;u+1;\frac{1}{2}\right)\right]}_{+} \end{array}$$

**Proof.**  Proof of Corollary 1 can be directly obtained via the successive application of the results derived in Theorems 1 and 2. □

#### 3.4.2. Exact and Asymptotic APD and AUC for the Beaulieu-Xie Shadowed Channel Model

For the Beaulieu-Xie shadowed fading channel model, the MGF is given by

(14) $$\begin{array}{c} M_{\gamma }^{\mathrm{BX}}\left(s\right)={\left(\frac{m_{Y}\Omega_{X}}{m_{X}\Omega_{Y}+m_{Y}\Omega_{X}}\right)}^{m_{Y}}{\left(\frac{m_{X}}{\Omega_{X}}\right)}^{m_{X}}\sum_{n=0}^{\infty }\frac{\Gamma (m_{Y}+n)}{n!\Gamma (m_{Y})}\times \\ \times {\left(\frac{m_{X}^{2}\Omega_{Y}}{\Omega_{X}\left(m_{X}\Omega_{Y}+m_{Y}\Omega_{X}\right)}\right)}^{n}{\left(\frac{m_{X}}{\Omega_{X}}-s\right)}^{-(m_{X}+n)}, \end{array}$$

with the parameters $m_{X},m_{Y},\Omega_{X},\Omega_{Y}$ defined as in [39].

**Lemma** **1.** *The moment-generating function of the Beaulieu-Xie shadowed channel model can be represented in the following form:*

(15) $$\begin{array}{c} M_{\gamma }^{\mathrm{BX}}\left(s\right)={\left(\frac{m_{Y}\Omega_{X}}{m_{X}\Omega_{Y}+m_{Y}\Omega_{X}}\right)}^{m_{Y}}{\left(\frac{m_{X}\left(\Omega_{X}+\Omega_{Y}\right)}{\bar{\gamma }\Omega_{X}}\right)}^{m_{X}}\times \\ \times {\left(\frac{m_{X}\left(\Omega_{X}+\Omega_{Y}\right)}{\bar{\gamma }\Omega_{X}}-s\right)}^{\;m_{Y}-m_{X}}\;\;{\left(\frac{m_{X}m_{Y}\left(\Omega_{X}+\Omega_{Y}\right)}{\bar{\gamma }\left(m_{X}\Omega_{Y}+m_{Y}\Omega_{X}\right)}-s\right)}^{-m_{Y}}\;\;\;\;\;\;\;. \end{array}$$

**Proof** **of** **Lemma** **1.** It should be mentioned that the MGF (14) is defined (see [39]) in terms of instantaneous power (not SNR, as for the abovementioned models), which is not suitable for further calculations, hence proper rescaling is needed. To do this, one can start with the envelope pdf $w_{R}\left(r\right)$ (see [39], Section III, Equation (7)) and defining the average power $E\left\{R^{2}\right\}=\left(\Omega_{X}+\Omega_{Y}\right)$ (by means of Equation (12) from [39]) to perform the change in variable $\gamma ≜\bar{\gamma }r^{2}/E\left\{R^{2}\right\}$, deriving the instantaneous SNR pdf as [4]: $w_{\gamma }\left(\gamma \right)=w_{R}\left(\sqrt{\frac{\gamma (\Omega_{X}+\Omega_{Y})}{\bar{\gamma }}}\right)/2\sqrt{\frac{\gamma \bar{\gamma }}{\left(\Omega_{X}+\Omega_{Y}\right)}}$. The latter part of the proof relies upon the series summation in (14) by using the identity $\sum_{n=0}^{\infty }\frac{\Gamma (n+m)}{n!\Gamma (m)}\alpha^{n}={\left(1-\alpha \right)}^{-m}$ and factorizing the terms containing argument s. □

**Corollary** **2.** *Based on Expression (15), the APD and AUC for this model are derived:*

(16) $$\begin{array}{c} {\bar{P}}_{D}^{\mathrm{BX}}=1-{\left(1+\frac{m_{X}}{m_{Y}}\frac{\Omega_{Y}\bar{\gamma }}{\left(m_{X}\left(\Omega_{X}+\Omega_{Y}\right)+\Omega_{X}\bar{\gamma }\right)}\right)}^{-m_{Y}}{\left(1+\frac{\Omega_{X}\bar{\gamma }}{m_{X}\left(\Omega_{X}+\Omega_{Y}\right)}\right)}^{-m_{X}}\frac{{\left(\frac{\lambda }{2}\right)}^{u}e^{-\frac{\lambda }{2}}}{\Gamma \left(u+1\right)}\times \\ \times \Phi_{2}^{\left(3\right)}\left(1,m_{X}-m_{Y},m_{Y};u+1;\frac{\lambda }{2},\frac{\lambda }{2}\left(\frac{\Omega_{X}\bar{\gamma }}{\left(\Omega_{X}\bar{\gamma }+m_{X}\left(\Omega_{X}+\Omega_{Y}\right)\right)}\right),\frac{\lambda }{2}\left(\frac{\left(m_{X}\Omega_{Y}+m_{Y}\Omega_{X}\right)\bar{\gamma }}{\left(m_{X}m_{Y}\left(\Omega_{X}+\Omega_{Y}\right)+\left(m_{Y}\Omega_{X}+m_{X}\Omega_{Y}\right)\bar{\gamma }\right)}\right)\right) \end{array}$$

(17) AUCBX=1−Γu+122πΓu+11+mXmYΩYγ¯(mX(ΩX+ΩY)+ΩXγ¯)−mY1+ΩXγ¯mX(ΩX+ΩY)−mX××FD(3)2u,1,mX−mY,mY;u+1;12,12ΩXγ¯(ΩXγ¯+mX(ΩX+ΩY)),12(mXΩY+mYΩX)γ¯(mXmY(ΩX+ΩY)+(mYΩX+mXΩY)γ¯)

*and their asymptotic versions are given by*

(18) $$\begin{array}{c} {\bar{P}}_{D\mathrm{as}}^{\mathrm{BX}}={\left[1-\frac{{\left(\frac{\lambda }{2}\right)}^{u}e^{-\frac{\lambda }{2}}}{\Gamma \left(u+1\right)}{\left(\frac{m_{X}}{m_{Y}}\frac{\Omega_{Y}\bar{\gamma }}{\left(m_{X}\left(\Omega_{X}+\Omega_{Y}\right)+\Omega_{X}\bar{\gamma }\right)}\right)}^{-m_{Y}}{\left(1+\frac{\Omega_{X}\bar{\gamma }}{m_{X}\left(\Omega_{X}+\Omega_{Y}\right)}\right)}^{-m_{X}}{}_{1}F_{1}\left(\mu +1;u+1;\frac{\lambda }{2}\right)\right]}_{+} \end{array}$$

(19) AUCasBX=1−Γu+122πΓu+11+ΩXγ¯mX(ΩX+ΩY)−mXmXmYΩYγ¯(mX(ΩX+ΩY)+ΩXγ¯)−mY2F12u,μ+1;u+1;12+

**Proof.**  Proof of Corollary 2 can be directly obtained via the successive application of the results derived in Theorems 1 and 2. □

### 3.5. Models’ Connections

The derived results for the generalized channel model with FPT MGF ((5) and (6)) and for the Fluctuating Beckmann ((10) and (11)) and Beaulieu-Xie shadowed models ((16) and (17)) and their asymptotic versions can help to gain a better insight into the physical meaning of the channel parameters and their connections with the known cases. First of all, one can see that the complexity of the obtained representation for the Beaulieu-Xie shadowed model is the same as for the κ−μ shadowed or η−μ model, or Hoyt model (in all the cases we observe the hypergeometric function of three variables), thus for these models to yield exactly the same results the parameters should be equal. This helps to establish the following chain of connections. Starting with the newest model (Beaulieu-Xie shadowed), one can connect it with the κ−μ shadowed in the following way:

(20) $$\begin{array}{c} m_{X}=\mu ,\;m_{Y}=m,\;\frac{\Omega_{Y}}{\Omega_{X}}=\kappa . \end{array}$$

Thus, one can view Beaulieu-Xie shadowed as an extended version of the κ−μ shadowed model since in the former one can handle $\Omega_{X}$ and $\Omega_{Y}$ independently and in the latter only their combination $\kappa =\frac{\Omega_{Y}}{\Omega_{X}}$. Thus, up to the substitutions (20), the results for the κ−μ shadowed model (derived via (5) and (6) with parameters defined in Section 3.2) and (16) and (17) are equivalent, hence one can gain a better understanding of how to reach the desired level of APD/AUC.

It can be easily noticed that by performing the following changes: $m=\mu ,\mu =2\mu ,\left(1+\kappa \right)=\left(h-H\right),\frac{1+\kappa }{1+2\kappa }=\left(h+H\right)$, one can connect the results for κ−μ shadowed and η−μ models. Finally, setting $\mu =\frac{1}{2}$, $\left(h-H\right)=\frac{1+q^{2}}{2}$, $\left(h+H\right)=\frac{1+q^{2}}{2q}$, one can relate the results for the Hoyt model to all of the abovementioned ones. It should be noted that, earlier, it was widely accepted [6] that the Hoyt model itself does not follow directly from the κ−μ shadowed model, but since the proposed expressions extend and unify those models into the FPT MGF one, the APD and AUC expressions are related up to the aforementioned substitutions.

## 4. Simulation and Results

To gain a better understanding of the energy-based spectrum sensing performance and the impact of various channel parameters upon the communication link quality for the two assumed models a comparative numerical analysis was carried out. The evaluation was performed for the case of arbitrary values of the parameter *u* (generally non-integer and non-half-integer, the cases that are usually addressed in literature). In order to conform with the existing studies, we assume the range of parameters to be as in [32] (for Fluctuating Beckmann) and [39] (for Beaulieu-Xie shadowed).

**Figure 1:**
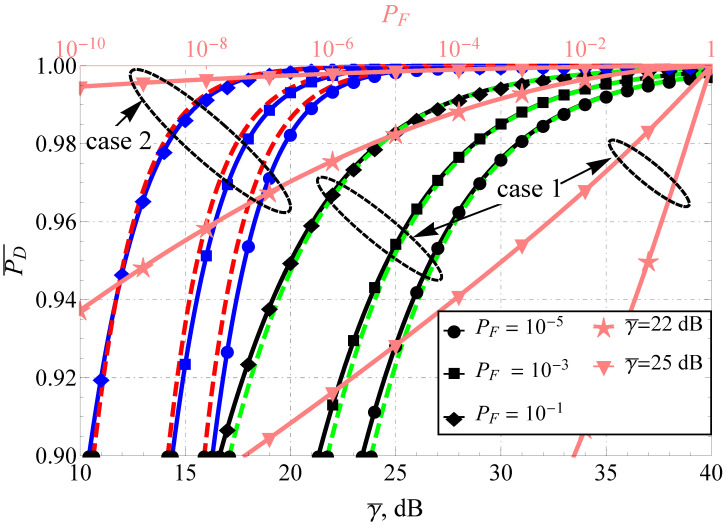
${\bar{P}}_{D}^{\mathrm{FB}}$ comparison for various $P_{D}$ and u=2.2: black lines (case 1)—κ=0.1, m=2, μ=1, η=0.1, $\rho^{2}=0.1$, blue lines (case 2)—κ=1, m=2, μ=2, η=10, $\rho^{2}=0.1$, dashed red and green lines—proposed approximations.

**Figure 2:**
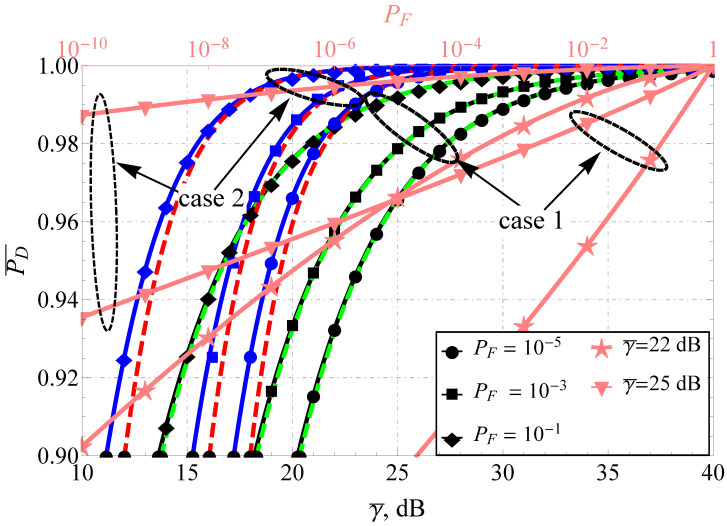
${\bar{P}}_{D}^{\mathrm{BX}}$ comparison for various $P_{D}$ and u=2.2: black lines (case 1)—$m_{X}=1$, $m_{Y}=2$, $\Omega_{X}=-1$ dB, $\Omega_{Y}=1$ dB, blue lines (case 2)—$m_{X}=2$, $m_{Y}=2$, $\Omega_{X}=1$ dB, $\Omega_{Y}=1$ dB, dashed red and green lines—proposed approximations.

The direct comparison of APD for the Fluctuating Beckmann and the Beaulieu-Xie shadowed models (see Figure 1 and Figure 2) demonstrates the difference in the performance for the high and low false alarm rate regions (which are common, for instance, in wireless cognitive networks). The increase in the number of multipath clusters and decrease in shadowing with some power imbalance of in-phase/quadrature components (ρ≠1) and LoS/NLoS components leads to the increase in APD. For both models, the improvement in SNR increases ${\bar{P}}_{D}$ as predicted, but the increment rate drastically differs. To demonstrate this, both plots are supplied with a second (upper) horizontal axis that depicts the functional dependence $P_{D}=f\left(P_{F}\right)$ (i.e., the average receiver operating characteristic) for two $\bar{\gamma }$ and two cases: poor propagation conditions (case 1) and improved conditions (case 2). Red and green dashed lines represent the derived approximating expressions. It can be seen that the approximations perform excellently for severe propagation conditions: high shadowing, a small number of multipath clusters and weak dominating components. Their quality degrades with the channel improvement but is still satisfactory (loses no more than 1 dB) for $\bar{\gamma }\geq 10$ dB, which constrains the effective applicability of the derived approximations.

It was established that for the Beaulieu-Xie shadowed model improvement in multipath component shadowing ($m_{X}=2$ instead of $m_{X}=1$), balancing the LoS/NLoS average power but downgrading the desired level of false alarm probability helps to maintain the same APD (for some SNR, e.g., $\bar{\gamma }=18$ dB, see the existing cross point).

One should note that the simulation parameters for the presented plots were chosen in such a way as to conform with the existing studies on the one hand and to incorporate the opposing fading scenarios on the other: heavy and light fading, with LoS/NLoS imbalance and without.

**Figure 3:**
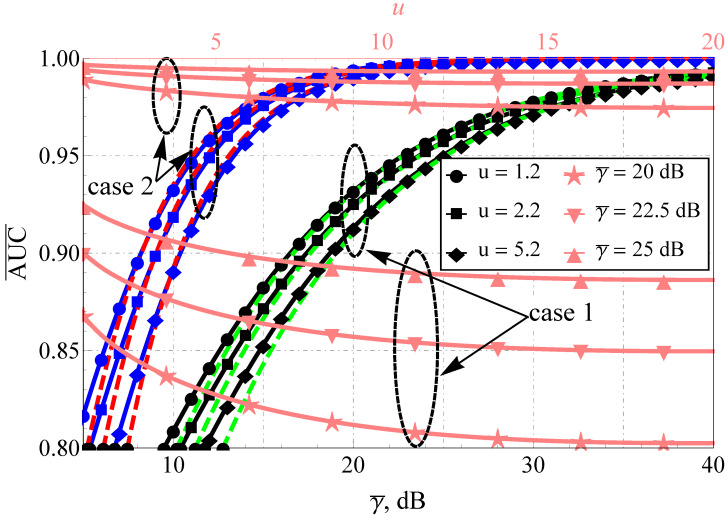
Average AUC comparison for Fluctuating Beckmann model and various u: black lines (case 1) show κ=0.1, m=0.5, μ=0.5, η=0.1, $\rho^{2}=0.1$, blue lines (case 2) show κ=10, m=1.5, μ=1, η=1, $\rho^{2}=0.1$, dashed red and green lines—proposed approximations.

**Figure 4:**
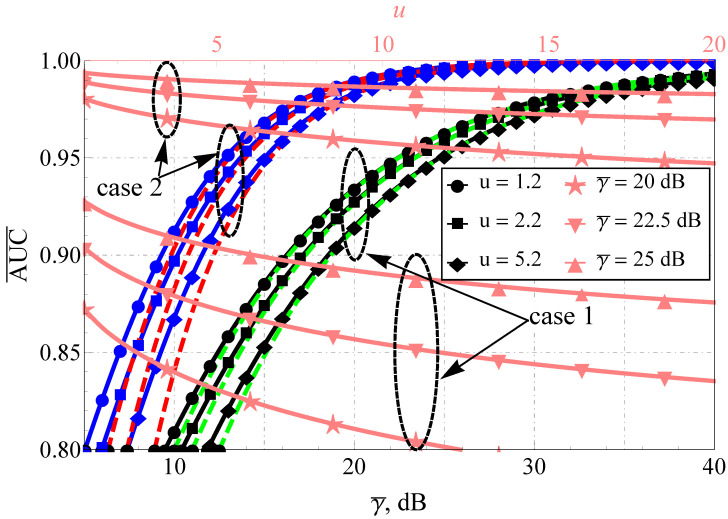
Average AUC comparison for Beaulieu-Xie shadowed model and various u: black lines (case 1) show $m_{X}=0.5$, $m_{Y}=0.1$, $\Omega_{X}=-1$ dB, $\Omega_{Y}=1$ dB, blue lines (case 2) show $m_{X}=1$, $m_{Y}=1$, $\Omega_{X}=-1$ dB, $\Omega_{Y}=-1$ dB, dashed red and green lines—proposed approximations.

As for the AUC, the improvement in the propagation conditions (see Figure 3 and Figure 4), as expected, increases the AUC for both models. However, again, the rate of AUC change differs with the increase in the sensing base (see the upper horizontal axis and the pink lines): for the Fluctuating Beckmann channel with good propagating conditions and u≥5 or bad conditions with u≥10, a further increase in u mostly does not impact AUC, but this is not the case for the Beaulieu-Xie shadowed model, where the AUC saturation region (in terms of u) shifts to the very high sensing bases. Here, one assumes a strong imbalance of the LoS/NLoS component power ratio with dominating LoS (blue lines) and NLoS (black lines) and different numbers of multipath clusters (0.5 with high shadowing versus 1 with smaller ones) for the Fluctuating Beckmann model. For the Beaulieu-Xie shadowed channel, the cases of the several LoS components (being a unique property of this model) and almost total blockage of LoS with $m_{Y}<0.5$ (a specific trait of (14)) were accounted for. The overall increase in the sensing base decreases the AUC values for both models but the rate of the decrease is different: the smaller power imbalance of LoS/NLoS components leads to a greater spread of the curves (κ=0.1 or 1 for Figure 3 and $\Omega_{X}=\Omega_{Y}=1$ dB or $\Omega_{X}=-1$ dB, $\Omega_{Y}=-1$ dB, which can be recalculated via (20) to κ=1.5 or 1 for Figure 4).

**Figure 5:**
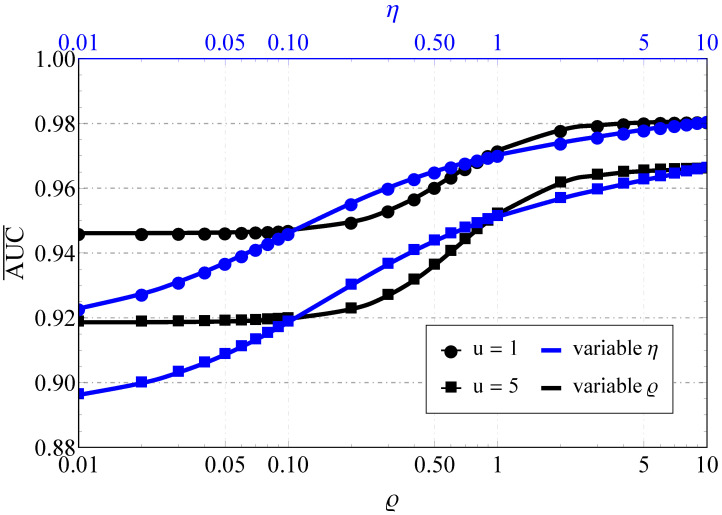
Average AUC comparison for the Fluctuating Beckmann (with κ=1, m=1, μ=1, $\bar{\gamma }=15$ dB): blue lines—variable η with $\rho^{2}=0.1$, black lines—variable *ρ* with η=0.1.

Figure 5 considers the impact of LoS (i.e., variable *ρ*) and NLoS (i.e., variable *η*) component imbalances on the Fluctuating Beckmann model and demonstrates that the increase in such an imbalance (regardless of the component type) actually improves AUC. One interesting effect that was found is the existence of AUC saturation regions (when ρ≥2 or ρ≤0.1), which practically means that it is insensitive to LoS in-phase/quadrature component balance fluctuations when one of them becomes highly predominant.

**Figure 6:**
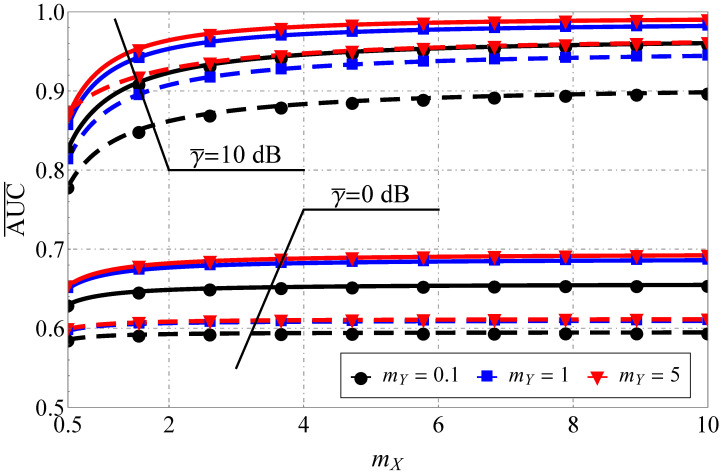
Average AUC comparison for the Beaulieu-Xie shadowed model for various $m_{X}$ and $\bar{\gamma }=10$ dB, $\Omega_{X}=1$ dB, $\Omega_{Y}=1$ dB: solid lines show u=1, dashed lines show u=5.

The analysis of AUC for the Beaulieu-Xie shadowed model depending on its parameters (see Figure 6) demonstrated that, in the case of small SNR and balanced LoS/NLoS components, the AUC is practically insensitive to the change in $m_{X}$ (for $m_{X}>1.5$) irrespective of $m_{Y}$ and u. Furthermore, the increase in $m_{Y}$ (for $m_{Y}>2$) does not introduce any sufficient gain in AUC. Thus, in the low-SNR regime, those values can be assumed as almost asymptotic (from a computational point of view). For the case of high SNR, this saturation region is extended and is highly dependent on the value of the sensing base, which introduces a pronounced impact upon the AUC. Moreover, the derived results can help to understand the possible adaptation strategies of the sensing procedure and signal processing: for instance, one can see that AUCs for the cases of $m_{Y}=5,u=5$ and $m_{Y}=0.1,u=1$ (for $m_{X}>1.5$) almost coincide, which means that the effects of the increase in LoS component shadowing can be balanced out by the appropriate reduction in the sensing base.

**Figure 7:**
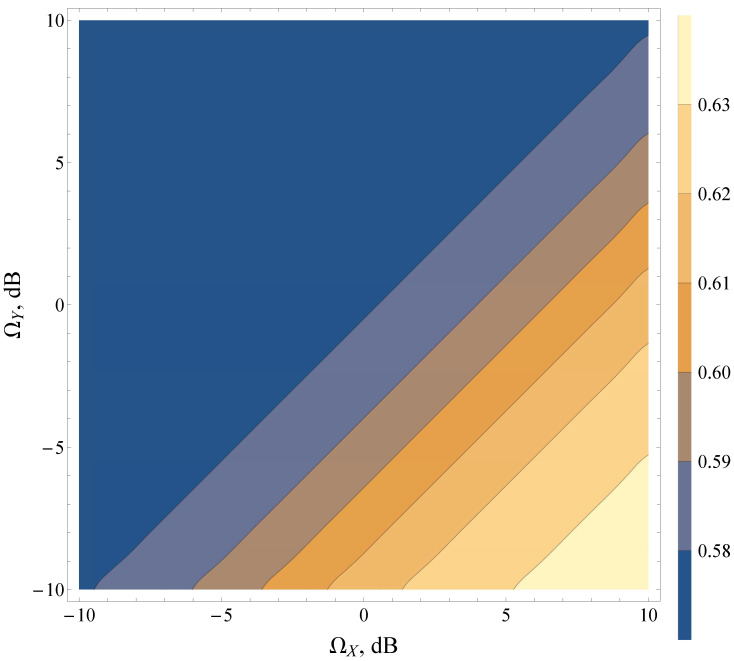
Average AUC contour map for the Beaulieu-Xie shadowed model for various $\Omega_{X},\Omega_{Y}$ and $\bar{\gamma }=0$ dB, u=2, $m_{X}=0.1$, $m_{Y}=1$.

**Figure 8:**
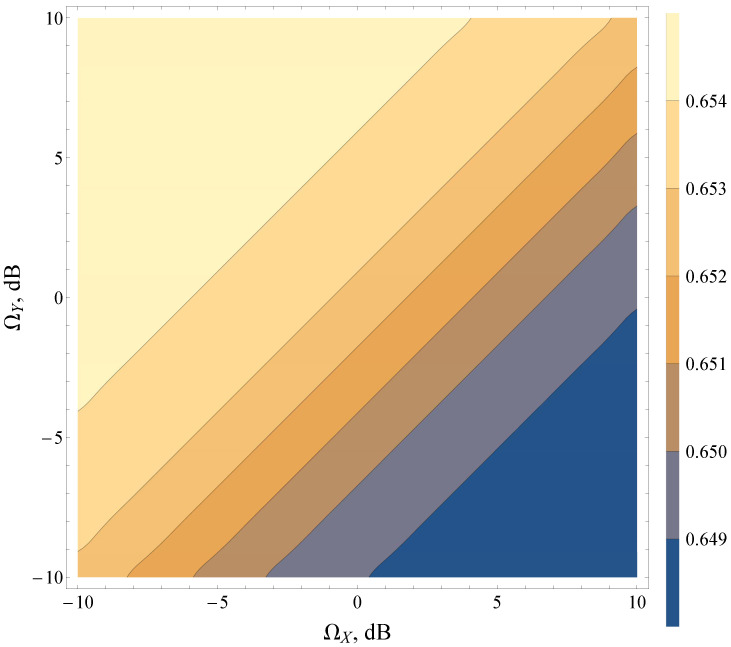
Average AUC contour map for the Beaulieu-Xie shadowed model for various $\Omega_{X},\Omega_{Y}$ and $\bar{\gamma }=0$ dB, u=2, $m_{X}=5$, $m_{Y}=2$.

It was found that the combination of various average powers of LoS/NLoS components (i.e., $\Omega_{Y}$ and $\Omega_{X}$) can lead to strictly antipodal behavior of the detection metrics. The research demonstrated that for heavy fading and shadowing conditions (both LoS and NLoS components) (see contour map in Figure 7), the increase in the total power of multipath waves (for a fixed power of dominant components) increases AUC, whereas, in the case of light fading with multiple LoS components (see contour map in Figure 8), the effects are exactly the opposite. Moreover, the lines of constant AUC (contour lines) can be approximated as $\Omega_{Y}=\Omega_{X}+b_{\mathrm{dB}}$, where $b_{\mathrm{dB}}$ is a constant shift in a log scale, representing LoS/NLoS component power imbalance.

## 5. Discussion and Further Generalization

It is trivial that the idea of channel model unification itself, due to the existing similarities in their MGF notation, is not novel, and as was mentioned earlier, the definition (4) was first introduced in [26] (see Definition 1 for a monomial/posynomial MGF). Though being exemplary research with a very high possible impact in various areas, it did not have any further elaboration. Moreover, independently of this work, this definition was revitalized in [41], that used a contour-integral approach proposed in [16] to analytically solve the problem of the average bit/symbol error rate calculation in the presence of fading, thus extending the results proposed in [26]. Since the approach in [16] (assumed herein) relies on a more general formulation, the Gaussian Q-function used in [26] is only a specific limiting case of a more general Marcum Q-function (used in [16] and herein); the presented results cannot be deduced from [26], and thus can be assumed as its generalization that expands the initial methodology.

Although the proposed FPT type of the MGF of the random fluctuating channel incorporates a wide range of NLoS and shadowed LoS models, it can be further extended. For instance, as assumed in this research, Gamma and κ−μ shadowed models were efficiently used to describe SIMO communication systems [42,43] (with multiple receivers) and systems with signal aggregation form several subbands (with single or multiple receivers) [44]. It can be easily verified that the MGFs for those cases are readily represented in form, given by (4), hence the derived representations (5) and (6) remain valid for those cases. Moreover, the increase in the scope of the derived results can be performed via the Mixture-Gamma model application, which can be successfully implemented to approximate a wide range of models that are not directly covered by the FPT representation (see, for instance, [11,45,46]).

Regardless of the fact that the closed-form solutions are obtained in terms of the multivariate hypergeometric functions, they are quite frequently used in wireless communications. Despite not being directly implemented in modern software packages (such as Matlab, Mathematica, Maple, etc.), their computation (efficiently performed by numerical calculation of the inverse Laplace transform, for example, exhaustively discussed in [47,48,49,50]), including truncation errors, and the required number of summands and achievable computational gain (relative to the numeric integration) are frequently discussed in the literature (see, for instance, [16,28,32,51,52]).

It should be noted that the present research claims that the FPT MGF representation can handle a wide range of NLoS and shadowed LoS models. This is not the case with the LoS situations, since in those cases MGFs include an exponential multiplier that is not directly in the form (4). At the same time, it is well known (see, for instance, [28,32,39]) that in the limiting cases (when the shadowing parameter goes to infinity) shadowed LoS models yield classical LoS ones, thus the results herein (for example, in the case of m→∞ for κ−μ shadowed and Fluctuating Beckmann and $m_{Y}\to \infty$ for Beaulieu-Xie shadowed) can handle a wider range of possible models. As an alternative approach, one can obtain the same limiting expressions by performing a limiting operation over the exponential multiplier in the MGF expression and rewriting it in the limiting form of (4), finally yielding the same expressions. Although some results are derived, specific questions on performing a limiting operation over the multivariable hypergeometric series arise with no closed-form analytical solution to date. Even though it was verified, evaluating the limits can be performed numerically (the regions of the parameter asymptotics derived herein are highly valuable for that procedure), the questions of numerical stability begin to play a crucial role, dominating over the numerical efficiency of the solution. For example, for a high signal-to-noise regime, the expansion coefficients of the respective multivariate hypergeometric series will prevent fast convergence, thus a large number of terms will be needed, slowing down the solution. Moreover, some questions about the fulfilment of the dominated convergence theorem, and the possibility of interchanging limiting and integration operation, arise. Thus, such an extension still remains a challenging open problem.

## 6. Conclusions

The present research studies the performance of energy-based detection in the presence of multipath fading and shadowing effects. The quality of the procedure is assessed in terms of such metrics as the average probability of detection and the area under the average receiver operating characteristic curve. To attempt a unification of the results for the existing channel models, a new generalization for the class of the fading channel moment-generating functions (factorized power-type MGF representation) is proposed. It is shown that such a formalism directly includes a wide range of existing classical and generalized models with non-line-of-sight and shadowed line-of-sight situations. The relations between the models are demonstrated. The derived general expressions are applied to the closed-form analysis of new generalized models: Fluctuating Beckmann and Beaulieu-Xie shadowed. The obtained closed-form solutions for the assumed quality metrics of those models are then numerically analyzed for various channel parameters.

## Data Availability

Not applicable.

## Appendix A. Proof of Theorem 1

**Proof.** To prove Theorem 1, one starts by combining (1) and (2) and applying the contour-integral representation of the generalized Marcum Q-function given in [16]:

(A1) $$\begin{array}{c} Q_{M}\left(a,b\right)=\frac{1}{2\pi i}e^{-\frac{a^{2}+b}{2}}\int_{C_{H}}\frac{e^{\frac{a^{2}}{2s}+\frac{b^{2}s}{2}}}{s^{M}\left(1-s\right)}ds \end{array}$$

with the integration Hankel-type contour $C_{H}$ starting from −∞ in the lower half-plane, running counterclockwise and encircling the origin and ending at −∞ in the upper half-plane. Combining (A1) and (2), changing the order of integration and applying the definition of the MGF yields:

(A2) $$\begin{array}{c} {\bar{P}}_{D}^{\mathrm{FPT}}=\frac{1}{2\pi i}e^{-\frac{\lambda }{2}}\int_{0}^{\infty }e^{-\gamma }\int_{C_{H}}\frac{e^{\frac{\gamma }{s}}e^{\frac{\lambda s}{2}}}{s^{u}\left(1-s\right)}dsw_{\gamma }\left(\gamma \right)d\gamma =\\ =\frac{1}{2\pi i}e^{-\frac{\lambda }{2}}\int_{C_{H}}\frac{e^{\frac{\lambda s}{2}}}{s^{u}\left(1-s\right)}\underset{M_{\gamma }\left(\frac{1}{s}-1\right)}{\underset{︸}{\int_{0}^{\infty }w_{\gamma }\left(\gamma \right)e^{\left(\frac{1}{s}-1\right)\gamma }d\gamma }}ds=\\ =\frac{1}{2\pi i}e^{-\frac{\lambda }{2}}\int_{C_{H}}\frac{M_{\gamma }\left(\frac{1}{s}-1\right)e^{\frac{\lambda s}{2}}}{s^{u}\left(1-s\right)}ds. \end{array}$$

Closing the contour of integration $C_{H}$ as is done in [16] (see Section 3, Figure 2), it is possible to represent APD in the following form:

(A3) P¯DFPT=Ap∏j=1Nδj(1+αj)βjeλ2ress=1s−(u+1)(1−1s)∏j=1N1−θjsβj−−L−1s−(u+1)(1−s−1)−1∏j=1N1−θjsβj,λ2

where $L^{-1}\left\{\cdot ,\frac{\lambda }{2}\right\}$ is the inverse Laplace transform evaluated at $\frac{\lambda }{2}$ and ${\mathrm{res}}_{s=1}\left\{\cdot \right\}$ is the residue at point s=1. Making use of [53] (Equation (54), Chapter 9, p. 290), calculating the residue and simplifying the expression accounting for the Lauricella confluent hypergeometric series of N+1 variables $\Phi_{2}^{\left(N+1\right)}\left(b_{1},\ldots ,b_{N+1};c;x_{1},\ldots ,x_{N+1}\right)$ (see [53] Equation (8), Chapter 1, p. 34) yields (5). To prove (6), one can notice that the integration over the $P_{F}$ can be interchanged with the integration over the threshold λ (for instance, see [12]), i.e.,

(A4) $$\begin{array}{c} \;{\mathrm{AUC}}^{\mathrm{FPT}}\;=\;\int_{0}^{1}\;{\bar{P}}_{D}^{\mathrm{FPT}}\;\left(P_{F}\right){\mathrm{dP}}_{F}\;=\;\int_{0}^{\infty }\;{\bar{P}}_{D}^{\mathrm{FPT}}\left(\lambda \right)\;\frac{\lambda^{u-1}e^{-\frac{\lambda }{2}}}{2^{u}\Gamma \left(u\right)}d\lambda \end{array}$$

Substituting (5) into (A4), applying the series representation of the Lauricella function (see [53] Equation (8), Chapter 1, p. 34) and changing the order of integration and summation yields

(A5) $$\begin{array}{c} {\mathrm{AUC}}^{\mathrm{FPT}}=A_{p}\prod_{j=1}^{N}\delta_{j}\alpha_{j}^{\beta_{j}}\left[1-\prod_{j=1}^{N}{\left(1+\frac{1}{\alpha_{j}}\right)}^{\beta_{j}}\frac{2^{-2u}}{\Gamma (u)\Gamma (u+1)}\times \right.\\ \times \sum_{l_{1}=0}^{\infty }\;\ldots \;\;\;\sum_{l_{N+1}=0}^{\infty }\;\;\;\frac{{\left(-\beta_{1}\right)}_{l_{1}}\;\ldots \;{\left(-\beta_{N}\right)}_{l_{N}}{\left(1\right)}_{l_{N+1}}}{{\left(u+1\right)}_{l_{\Sigma }}}\frac{{\left(\frac{1}{1+\alpha_{1}}\right)}^{l_{1}}\;\;\ldots \;{\left(\frac{1}{1+\alpha_{N}}\right)}^{l_{N}}}{l_{1}!\ldots l_{N+1}!}\left.\frac{1}{2^{l_{\Sigma }}}\int_{0}^{\infty }\lambda^{2u+l_{\Sigma }-1}e^{-\lambda }d\lambda \right] \end{array}$$

where $l_{\Sigma }=l_{1}+\ldots +l_{N+1}$ and ${\left(\cdot \right)}_{l}$ is the Pochhammer function. The last integral equals $\Gamma \left(2u\right){\left(2u\right)}_{l_{\Sigma }}$. Thus, collecting the terms and making use of the definition of the Appell series $F_{D}^{\left(N+1\right)}\left(a;b_{1},\ldots ,b_{N+1};c;x_{1},\ldots ,x_{N+1}\right)$ (see [53] Equation (4), Chapter 1, p. 33) finalizes the proof. □

## Appendix B. Proof of Theorem 2

**Proof.** To prove Theorem 2, one can note that

(A6) $$\begin{array}{c} \Phi_{2}^{\left(N+1\right)}\left(1,-\beta_{1},\ldots ,-\beta_{N};u+1;\frac{\lambda }{2},\frac{\lambda /2}{1+\alpha_{1}},\ldots ,\frac{\lambda /2}{1+\alpha_{N}}\right)=\\ ={\left(\frac{\lambda }{2}\right)}^{-u}\Gamma \left(u+1\right)L^{-1}\left\{\frac{s^{-(u+1)}}{\left(1-s^{-1}\right)}\prod_{j=1}^{N}{\left(1-\frac{\frac{1}{1+\alpha_{j}}}{s}\right)}^{\beta_{j}},\frac{\lambda }{2}\right\} \end{array}$$

Assuming that the high-SNR regime (i.e., γ→∞) constitutes the case when all $\alpha_{i}\to 0$ and taking the limit of both sides of (A6) yields:

(A7) $$\begin{array}{c} {\left(\frac{\lambda }{2}\right)}^{-u}\Gamma \left(u+1\right)L^{-1}\left\{s^{-(u+1)}{\left(1-s^{-1}\right)}^{-\sum_{j=1}^{N}\beta_{j}+1},\frac{\lambda }{2}\right\}={}_{1}F_{1}\left(1-\sum_{i=1}^{N}\beta_{i};u+1;\frac{\lambda }{2}\right), \end{array}$$

which completes the first part of the proof. To derive (8), one can use the integral relation between Lauricella and Appell functions (see [53]):

(A8) $$\begin{array}{c} F_{D}^{\left(N+1\right)}\left(2u;1,-\beta_{1},\ldots ,-\beta_{N};u+1;\frac{1}{2},\frac{1/2}{1+\alpha_{1}},\ldots ,\frac{1/2}{1+\alpha_{N}}\right)=\\ =\frac{1}{\Gamma (2u)}\int_{0}^{\infty }e^{-t}t^{2u-1}\Phi_{2}^{\left(N+1\right)}\left(1,-\beta_{1},-\beta_{N};u+1;\frac{t}{2},\frac{t/2}{1+\alpha_{1}},\ldots ,\frac{t/2}{1+\alpha_{N}}\right)dt \end{array}$$

Applying the result (A7), one obtains

(A9) $$\begin{array}{c} \lim_{\alpha_{i}\to 0,\forall i}F_{D}^{\left(N+1\right)}\left(\cdot \right)=\frac{1}{\Gamma (2u)}\int_{0}^{\infty }e^{-t}t^{2u-1}{}_{1}F_{1}\left(1-\sum_{i=1}^{N}\beta_{i};u+1;\frac{t}{2}\right)dt. \end{array}$$

The last integral can be regarded as the Laplace transform evaluated at 1, thus one has:

(A10) $$\begin{array}{c} L^{-1}\left\{t^{2u-1}{}_{1}F_{1}\left(1-\sum_{i=1}^{N}\beta_{i};u+1;\frac{t}{2}\right),1\right\}={}_{2}F_{1}\left(2u;1-\sum_{i=1}^{N}\beta_{i};u+1;\frac{1}{2}\right). \end{array}$$

Lastly, it should be noted that the derived asymptotics do not preserve the positiveness of the APD and AUC, thus the expressions should be supplemented with the positive-part function. □
